# The room temperature preservation of filtered environmental DNA samples and assimilation into a phenol–chloroform–isoamyl alcohol DNA extraction

**DOI:** 10.1111/1755-0998.12281

**Published:** 2014-06-11

**Authors:** Mark A Renshaw, Brett P Olds, Christopher L Jerde, Margaret M McVeigh, David M Lodge

**Affiliations:** * Department of Biological Sciences, University of Notre Dame100 Galvin Life Sciences Center, Notre Dame, IN, 46556, USA; † Unit 117, Environmental Change Initiative1400 East Angela Boulevard, South Bend, IN, 46617, USA

**Keywords:** eDNA extraction, eDNA preservation, environmental DNA, *Lepomis macrochirus*

## Abstract

Current research targeting filtered macrobial environmental DNA (eDNA) often relies upon cold ambient temperatures at various stages, including the transport of water samples from the field to the laboratory and the storage of water and/or filtered samples in the laboratory. This poses practical limitations for field collections in locations where refrigeration and frozen storage is difficult or where samples must be transported long distances for further processing and screening. This study demonstrates the successful preservation of eDNA at room temperature (20 °C) in two lysis buffers, CTAB and Longmire's, over a 2-week period of time. Moreover, the preserved eDNA samples were seamlessly integrated into a phenol–chloroform–isoamyl alcohol (PCI) DNA extraction protocol. The successful application of the eDNA extraction to multiple filter membrane types suggests the methods evaluated here may be broadly applied in future eDNA research. Our results also suggest that for many kinds of studies recently reported on macrobial eDNA, detection probabilities could have been increased, and at a lower cost, by utilizing the Longmire's preservation buffer with a PCI DNA extraction.

## Introduction

The detection of macrobial DNA in environmental water samples, hereafter referred to as ‘eDNA’, is a burgeoning field of research often involving the detection of rare species, including invasive species (Dejean *et al*. 2012; Goldberg *et al*. 2013; Jerde *et al*. 2013; Takahara *et al*. 2013; Piaggio *et al*. 2014) and endangered species (Olson *et al*. 2012; Thomsen *et al*. 2012a). Three important considerations for eDNA research are the capture of the eDNA, the preservation of the eDNA and the successful extraction of the eDNA (Pilliod *et al*. 2013). The filtration of water samples is a routine capture mechanism of eDNA present in aquatic environments as it is scalable to the environment and allows for the concentration of rare eDNA fragments from large volumes of water. Various filter membrane types and filter pore sizes have been utilized for the filtration of water samples in eDNA studies (Minamoto *et al*. 2012; Thomsen *et al*. 2012b; Goldberg *et al*. 2013; Jerde *et al*. 2013; Piaggio *et al*. 2014). These choices can impact the efficiency of the eDNA capture (Liang & Keeley 2013), but are currently difficult to quantify between studies as other aspects (i.e. eDNA preservation and extraction) are not held constant (Turner *et al*. 2014).

For studies utilizing filtration of water samples, the preservation of eDNA is often reliant on cold ambient temperatures (Mahon *et al*. 2010; Takahara *et al*. 2012, 2013; Jerde *et al*. 2013; Wilcox *et al*. 2013). This is the case at multiple points of the sample collection process, including the use of ice in the transport of water samples from collection sites to laboratories, storage of water samples in freezers for filtering at later time points and freezing of filters following the processing of water samples (Mahon *et al*. 2010; Thomsen *et al*. 2012b; Pilliod *et al*. 2013; Takahara *et al*. 2013; Wilcox *et al*. 2013; Piaggio *et al*. 2014). This reliance on cold ambient temperatures poses practical limitations for field collections in locations where refrigeration and frozen storage is difficult (i.e. backcountry locations accessible only by foot) or where samples must be transported long distances for further processing and screening (i.e. international travel). The justification for such temperature control is to limit DNA degradation in eDNA samples, a concern of paramount importance in eDNA studies as the targeted fragments are often degraded at the time of collection and/or are present in vanishing amounts, due to the rarity of the organisms producing the eDNA. While *in situ* field filtration can be overcome with portable pumps (Pilliod *et al*. 2013; Wilcox *et al*. 2013), having a method that preserves filtered eDNA and prevents further degradation would benefit field scientists tasked with collecting samples under transport or temperature limitations.

The storage of filters in ethanol is a current room temperature preservation option (Goldberg *et al*. 2013; Pilliod *et al*. 2013), but the ethanol is not itself incorporated into the DNA extraction process. A number of preservation buffers, in addition to ethanol, have shown to be effective with tissue samples at room temperature (Seutin *et al*. 1991; Longmire *et al*. 1997; Kilpatrick 2002; Rhodes *et al*. 2003) and should serve in a similar capacity with eDNA captured on filters. Many of these room temperature preservation buffers simultaneously facilitate the lysis of cellular membranes, releasing intracellular components, such as DNA, into the preservation buffer. For eDNA studies, the assimilation of a room temperature preservation buffer into a DNA extraction protocol would increase the efficiency of the extraction (i.e. include eDNA that washes off the filter prior to extraction or eDNA released through lysis of cellular membranes) while avoiding hassles related to the storage of samples at a cold ambient temperature (Seutin *et al*. 1991; Kilpatrick 2002).

The extraction of filtered eDNA is often accomplished with a commercial kit, such as MoBio's PowerWater® DNA Isolation kit (Olson *et al*. 2012; Jerde *et al*. 2013; Wilcox *et al*. 2013; Piaggio *et al*. 2014) or Qiagen's DNeasy® Blood and Tissue kit (Minamoto *et al*. 2012; Goldberg *et al*. 2013; Pilliod *et al*. 2013; Kelly *et al*. 2014). Phenol–chloroform–isoamyl alcohol DNA extractions (Sambrook *et al*. 1989), hereafter ‘PCI’, are a popular extraction technique utilized in conjunction with room temperature preservation buffers for tissue samples in various applications of conservation genetics (Miller 2006; Smith & Hughes 2008; Wirgin *et al*. 2010). Additionally, this protocol has been recently employed for the extraction of macrobial eDNA captured on polycarbonate track-etch filters, nylon filters and glass fibre filters (Barnes *et al*. 2014; Deiner & Altermatt 2014; Turner *et al*. 2014). The PCI extraction protocol has the potential to drastically reduce per sample costs currently associated with eDNA research and assimilate a room temperature preservation buffer into the extraction process, integrating a transport and storage mechanism that does not rely on cold ambient temperatures.

As eDNA projects currently employ a variety of capture, preservation and extraction protocols (Lodge *et al*. 2012; Pilliod *et al*. 2013), some standardization could help with comparisons of other aspects of the research that may be heavily influenced by environmental conditions, such as the use of various filter membrane types and pore sizes in the capture of targeted eDNA fragments (Barnes *et al*. 2014; Turner *et al*. 2014). The room temperature preservation would additionally allow for application in conditions not suited for cold storage of samples. With these considerations in mind, we conducted a set of four experiments to compare (i) preservation with CTAB and Longmire's buffers among fresh samples, and samples stored for 1 and 2 weeks at −20, 20 and 45 °C, (ii) the application of the PCI protocol for eDNA extraction from cellulose nitrate filters, polyethersulfone filters, polycarbonate track-etch filters and glass microfibre filters (iii) the PCI DNA extraction protocol with two commercial DNA extraction kits currently featured in eDNA research and (iv) different approaches to the PCI DNA extraction protocol.

## Materials and methods

Separate rooms were used for fish husbandry, pre-PCR laboratory work and post-PCR laboratory work. The 70-gallon mesocosm, with approximately 100 juvenile bluegill (*Lepomis macrochirus*), was monitored throughout the experiment following institutional animal care and use protocols. For all the four experiments, 250 mL water samples were collected and filtered immediately through a single filter; unless otherwise specified, DNA extractions immediately followed the terminus of sample filtration for each experiment. Samples were filtered with 47-mm magnetic filter funnels (Pall), and each filter funnel was completely immersed in 10% bleach for a minimum of 10 min and thoroughly rinsed with DI water prior to any subsequent filtration. A single sample from each experimental treatment was filtered before filtering a second sample from each experimental treatment and then a third and so on, randomly spreading the filtering effort across all experimental treatments and minimizing the potential impact of contamination on any one experimental treatment. Unless otherwise specified, the filters were placed in 2-mL microcentrifuge tubes and completely immersed in 900 *μ*L of CTAB buffer (1.4 M NaCl, 2% (w/v) cetyltrimethyl ammonium bromide, 100 mM Tris, 20 mM EDTA and 0.25 mM polyvinylpyrrolidone; Coyne *et al*. 2005). The CTAB buffer was chosen as it has been used successfully for ongoing research (Barnes *et al*. 2014; Turner *et al*. 2014). Unless otherwise specified, DNA extractions followed a modified PCI extraction and ethanol precipitation (Sambrook *et al*. 1989): (i) the 2-mL microcentrifuge tubes (filters and preservation buffer) were incubated in a 65 °C water bath for 10 min; (ii) 900 *μ*L of PCI (one phase, 25:24:1, Amresco) was added to each tube, and samples were vortexed for 5 seconds. The addition of chloroform disintegrates polycarbonate track-etch (hereafter ‘PCTE’) and polyethersulfone (hereafter ‘PES’) filter membranes (Stark *et al*. 1998; Turner *et al*. 2014), and as such, extra care was given to mix the liquid layers for the cellulose nitrate (hereafter ‘CN’) and glass microfibre (hereafter ‘GMF’) filter membrane types, which remained intact; (iii) tubes were centrifuged at 15 000 ***g*** for 5 min, and 700 *μ*L of the aqueous layer was transferred to a fresh set of 2-mL microcentrifuge tubes; (iv) 700 *μ*L of chloroform–isoamyl alcohol, hereafter ‘CI’ (24:1, Amresco), was added to each tube and samples were vortexed for 5 seconds; (v) tubes were centrifuged at 15 000 ***g*** for 5 min, and 500 *μ*L of the aqueous layer was transferred to a fresh set of 2 mL tubes; (vi) 1.25 mL of 100% ice-cold ethanol and 20 *μ*L of 5 M NaCl were added to each tube, and samples were precipitated at −20 °C overnight; (vii) the precipitate was pelleted by centrifugation at 15 000 ***g*** for 10 min, and the liquid was decanted; (viii) pellets were dried in a vacuufuge at 45 °C for 15 min, followed by air drying until no visible liquid remained; and finally, (ix) pellets were rehydrated with 200 *μ*L of 1× TE Buffer, low EDTA (USB).

All DNA extractions were assayed with qPCR TaqMan® primers and probe targeting a 100-bp fragment of the bluegill cytochrome b gene (Takahara *et al*. 2013) in the following 20 *μ*L mixes: 10 *μ*L of TaqMan® Environmental Master Mix 2.0 (Life Technologies), 1.8 *μ*L of each primer (10 *μ*M stock concentration), 0.25 *μ*L of the TaqMan® probe (10 *μ*M stock concentration), 4 *μ*L of eDNA extract and 2.15 *μ*L of sterile water. The cycling parameters were as follows: a single step at 50 °C for 2 min, a single step at 95 °C for 10 min and 55 cycles at 95 °C for 15 seconds followed by 60 °C for 1 min. To quantify the DNA copy number in each eDNA extract, a standard was created (as follows) and included on each qPCR plate along with the eDNA extracts. A DNA fragment was synthesized by Integrated DNA Technologies based on the sequence from GenBank Accession no. JN389795 starting at location 14 298 and ending at location 14 797. The 500-bp fragment included the 100-bp region of the bluegill cytochrome b gene targeted by the assay flanked by an additional 200-bp on either side. The copy number of the synthesized standard was determined by multiplying the number of moles by Avogadro's number. A serial dilution of the standard was run on each qPCR plate and provided a regression line from which the unknown copy numbers of the eDNA extracts could be estimated. All qPCR assays were run on a Mastercycler ep realplex real-time PCR system (Eppendorf) and analysed with the accompanying realplex 2.2 software. Two negative controls were included on each qPCR plate, both containing the aforementioned 20 *μ*L mix except for additional sterile water in place of eDNA extract.

### Filter preservation experiment

A pilot experiment evaluated the use of four storage buffers: a 20% DMSO buffer (20% DMSO, 0.25M EDTA, saturated with NaCl; Seutin *et al*. 1991), RNAlater (Qiagen #76106), CTAB buffer and Longmire's buffer (0.1 M Tris, 0.1 M EDTA, 10 mM NaCl, 0.5% (w/v) SDS; Longmire *et al*. 1997). The 20% DMSO and RNAlater were not compatible with the PCI DNA extraction protocol as they both yielded a substantial precipitate that appeared to completely inhibit qPCR amplification. The removal of the 20% DMSO and RNAlater immediately prior to the DNA extraction and replacement with CTAB reduced the resulting precipitate, but the qPCR assays again failed to amplify. As such, only the CTAB buffer and Longmire's buffer were evaluated in the filter preservation experiment.

For each experimental treatment, five 250 mL water samples were each immediately filtered through a single PCTE filter (1.2 *μ*m; Millipore). Filters were placed in 2-mL microcentrifuge tubes and completely immersed in 900 *μ*L of either CTAB buffer (35 samples total) or Longmire's buffer (25 samples total). For each storage buffer, a set of five filters was extracted immediately, hereafter ‘fresh’. For the CTAB, an additional 10 filters were kept at each of the three temperature regimes: −20, 20 and 45 °C. For each temperature regime, 5 of the filters were extracted after a 1-week storage period, while the remaining 5 were extracted after a 2-week storage period. For the Longmire's buffer, the same protocol was applied for only two of the temperature regimes, 20 and 45 °C. The −20 °C regime was only used with the CTAB storage buffer as this has been used with success in the past (Barnes *et al*. 2014; Turner *et al*. 2014) and served as a benchmark against which the other temperature regimes and storage buffer could be compared. All DNA extracts from a given storage buffer were assayed once simultaneously on the same qPCR plate with a serial dilution of the standard for the quantification of DNA copy number. Each plate was then run a second time to produce two qPCR replicates for each sample.

### Filter membrane type experiment

The feasibility of DNA extraction from different filter membrane types with the PCI extraction protocol was evaluated for four different filter membrane types: 0.8 *μ*m CN (Whatman), 0.8 *μ*m PES (Pall), 1.0 *μ*m PCTE (GE Osmonics) and 1.5 *μ*m GMF (Whatman). For each filter membrane type, ten 250 mL water samples were each filtered through a single filter and completely immersed with 900 *μ*L of CTAB in a 2-mL microcentrifuge tube. DNA was extracted and ethanol precipitated with the previously described PCI protocol. All of the samples (in duplicate) were run simultaneously on a single qPCR plate with a serial dilution of the standard for the quantification of DNA copy number.

### PCI kit comparison experiment

Two historically popular combinations of macrobial eDNA filtration membrane types with commercial kit eDNA extractions have utilized 0.45-*μ*m CN filters with Qiagen's DNeasy® Blood and Tissue kit (Ahmed *et al*. 2010, 2013; Goldberg *et al*. 2011, 2013; Pilliod *et al*. 2013, 2014) and 1.5-*μ*m GMF filters with MoBio's PowerWater® DNA Isolation kit (Olson *et al*. 2012; Jerde *et al*. 2013; Wilcox *et al*. 2013; Piaggio *et al*. 2014). As such, comparisons between the PCI DNA extraction protocol and these two commercial DNA extraction kits focused on the same pairings of filters and extraction kits. A total of forty 250 mL water samples were collected, and twenty of them were each filtered immediately through a single 0.45-*μ*m CN filter (Spectrum). Ten of the CN filters were completely immersed in 900 *μ*L of CTAB, incubated in a 65 °C water bath for 1 h and then put through the PCI extraction and ethanol precipitation protocol as outlined previously. DNA was extracted from the remaining 10 CN filters following Qiagen's recommendations for the DNeasy® Blood and Tissue kit, with some modifications. Filters were completely immersed in 567 *μ*L buffer ATL and 63 *μ*L Proteinase-K (rather than the recommended 180 and 20 *μ*L, respectively) and incubated in a 65 °C water bath for 1 h. Following the incubation time, 630 *μ*L buffer AL and 630 *μ*L 100% ethanol were added to the 2-mL tube, instead of the recommended 200 *μ*L of each solution. A total of three centrifugation iterations were required to load the entire contents of the 2-mL tube, minus the CN filter, onto the silica membrane, as compared to the single centrifugation step normally required. The remainder of the protocol followed the manufacturer's recommendations. The other 20 samples were each filtered immediately through a single 1.5 *μ*m GMF filter (Whatman). Ten of the GMF filters were completely immersed in 900 *μ*L of CTAB, incubated in a 65 °C water bath for 1 h and then put through the PCI extraction and ethanol precipitation protocol as outlined previously. DNA was extracted from the remaining 10 GMF filters following MoBio's recommendations for the PowerWater® DNA Isolation kit, with the exception that the bead-beating step was performed until the filters appeared to be completely liquefied rather than the 5 min recommended maximum. DNA extractions from both kits were eluted from their respective spin columns with the addition of 200 *μ*L of 1× TE Buffer, low EDTA (USB), rather than the recommended Buffer AE (Qiagen) and Solution PW6 (MoBio). All of the samples (in duplicate) were run simultaneously on a single qPCR plate with a serial dilution of the standard for the quantification of DNA copy number.

### DNA extraction experiment

Variations on the PCI extraction protocol were evaluated, utilizing a total of forty 250 mL water samples each filtered immediately through a single 1.2-*μ*m PCTE filter (Millipore). Twenty of the filters were placed in 2-mL tubes with 900 *μ*L of CTAB, and DNA was extracted with the PCI protocol. An alternate precipitation protocol was evaluated using isopropyl alcohol with 10 of the samples in conjunction with the previously mentioned ethanol precipitation on the remaining 10 samples. The isopropyl alcohol precipitation was conducted as follows: (i) 500 *μ*L of ice-cold isopropyl alcohol and 250 *μ*L of 5M NaCl were added to the 500 *μ*L recovered from the aqueous layer (step 5 in the PCI protocol), and tubes were precipitated at −20 °C overnight; (ii) the precipitate was pelleted by centrifugation at 15 000 ***g*** for 10 min, and the liquid was decanted; (iii) 150 *μ*L of room temperature 70% ethanol was added to each tube; (iv) tubes were centrifuged at 15 000 ***g*** for 5 min, and the liquid was decanted; (v) 150 *μ*L of room temperature 70% ethanol was added to each tube a second time; (vi) tubes were centrifuged at 15 000 ***g*** for 5 min, and the liquid was decanted; (vii) pellets were dried in a vacuufuge at 45 °C for 15 min, followed by air drying until no visible liquid remained; and finally, (viii) pellets were rehydrated with 200 *μ*L of 1× TE Buffer, low EDTA (USB).

An alternate DNA extraction protocol eliminated the use of phenol (step 2 in the PCI protocol). The remaining 20 filters were completely immersed with 700 *μ*L of CTAB in 2-mL microcentrifuge tubes and incubated in a 65 °C water bath for 10 min. These samples were then extracted starting with the addition of 700 *μ*L of CI (step 4 in the PCI protocol). The ethanol and isopropanol precipitations were again both evaluated, each on 10 of the samples. All of the samples (in duplicate) were run simultaneously on a single qPCR plate with a serial dilution of the standard for the quantification of DNA copy number.

### Statistical analyses

ANOVA statistical tests were conducted individually for each of the four experiments to test for differences between mean DNA copy numbers. A two-sided *t*-test was used to test differences in the average amount of DNA recovered from fresh CTAB and Longmire's extractions within the ‘filter preservation experiment’. Technical replicates were averaged for the analysis, residuals from the ANOVAs and *t*-test were checked for normality using normal Q–Q plots, and pairwise comparisons in the ANOVA were performed using Tukey's post hoc test. All statistics and plots were conducted and created in Mathematica 9.0.1.0 (Wolfram Research, Inc., Version 9.0.1.0, Champaign, IL 2013). All tests conformed to the normality assumptions unless otherwise indicated.

## Results

For all of the qPCR plates run for this study, the qPCR standard curve slope ranged from −3.342 to −3.498, the y-intercept ranged from 38.43 to 39.26, the efficiency ranged from 0.93 to 0.99, and the *R*^2^ values ranged from 0.994 to 1.000. All of the negative controls failed to amplify throughout the entire experiment.

### Filter preservation experiment

**Fig 1:**
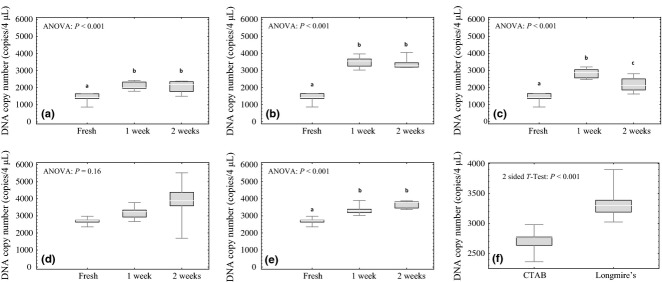
Box and whisker plots for the *filter preservation experiment*. The top and bottom of the whiskers represent the maximum and minimum values, the top and bottom of the boxes represent the 75% and 25% quartiles, and the lines inside the boxes represent the median values. Significance in pairwise comparisons of treatments is noted by letters a, b and c where different letters represent statistically significant differences. Two preservation buffers, CTAB and Longmire's, were evaluated over a 2-week interval of time. (a) CTAB with −20 °C storage, (b) CTAB with 20 °C storage, (c) CTAB with 45 °C storage, (d) Longmire's with 20 °C storage, (e) Longmire's with 45 °C storage and (f) comparison between CTAB and Longmire's for fresh extractions.

All replicates amplified and were incorporated into the statistical analyses for all twelve of the experimental treatments (Table1). For the CTAB preservation buffer, relative to fresh samples, Tukey's post hoc comparisons of the ANOVA results revealed a significantly higher DNA copy number in samples stored at all the three temperatures (−20, 20 and 45 °C) following the 2-week time interval (Fig.1a–c). For the Longmire's preservation buffer, the same result was observed for the 45 °C temperature (Fig.1e), but no significant difference in copy number existed between fresh samples and those stored at 20 °C (Fig.1d). A two-sided *t*-test of the fresh extractions revealed a significantly higher yield in DNA copy number for the Longmire's preservation buffer as compared to the CTAB preservation buffer (*P*-value < 0.001; Fig.1f).

**Table 1:** Outline of all four experiments, with the treatments evaluated in each experiment (Treatment) and the number of samples analysed per experimental treatment (*N*)

Experiment	Treatment	*N*
Filter preservation	CTAB; fresh	5
	CTAB; −20 °C; 1 week	5
	CTAB; −20 °C; 2 weeks	5
	CTAB; 20 °C; 1 week	5
	CTAB; 20 °C; 2 weeks	5
	CTAB; 45 °C; 1 week	5
	CTAB; 45 °C; 2 weeks	5
	Longmire's; fresh	5
	Longmire's; 20 °C; 1 week	5
	Longmire's; 20 °C; 2 weeks	5
	Longmire's; 45 °C; 1 week	5
	Longmire's; 45 °C; 2 weeks	5
Filter membrane type	0.8 *μ*m; cellulose nitrate (CN)	10
	0.8 *μ*m; polyethersulfone (PES)	10
	1 *μ*m; polycarbonate track-etch (PCTE)	10
	1.5 *μ*m; glass microfibre (GMF)	9
PCI kit comparison	0.45 *μ*m; cellulose nitrate (CN); PCI	10
	0.45 *μ*m; cellulose nitrate (CN); Qiagen	10
	1.5 *μ*m; glass microfibre (GMF); PCI	10
	1.5 *μ*m; glass microfibre (GMF); MoBio	10
DNA extraction	PCI start; ethanol precipitation	10
	PCI start; isopropanol precipitation	10
	CI start; ethanol precipitation	10
	CI start; isopropanol precipitation	10

### Filter membrane type experiment

**Fig 2:**
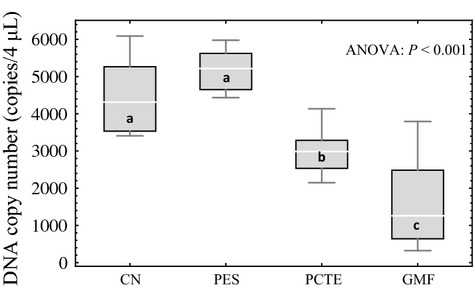
Box and whisker plots for the *filter membrane type experiment*. The top and bottom of the whiskers represent the maximum and minimum values, the top and bottom of the boxes represent the 75% and 25% quartiles, and the lines inside the boxes represent the median values. Significance in pairwise comparisons of treatments is noted by letters a, b and c where different letters represent statistically significant differences between experimental treatments. The four treatments were 0.8-*μ*m cellulose nitrate filters (CN), 0.8-*μ*m polyethersulfone filters (PES), 1.0-*μ*m polycarbonate track-etch filters (PCTE), and 1.5-*μ*m glass microfibre filters (GMF).

For three of the four filter membrane types (CN, PES and PCTE), all 10 samples amplified; one of the 10 samples for the GMF membrane type failed to amplify, and as such, only nine of the samples were used in the statistical analyses (Table1). Tukey's post hoc comparisons of the ANOVA results revealed that the 0.8-*μ*m CN and 0.8-*μ*m PES filters did not differ significantly and both yielded significantly more copies of DNA than the 1.0-*μ*m PCTE and 1.5-*μ*m GMF filters; the 1.0-*μ*m PCTE filters yielded significantly more copies of DNA than the 1.5-*μ*m GMF filters (Fig.2).

### PCI kit comparison experiment

**Fig 3:**
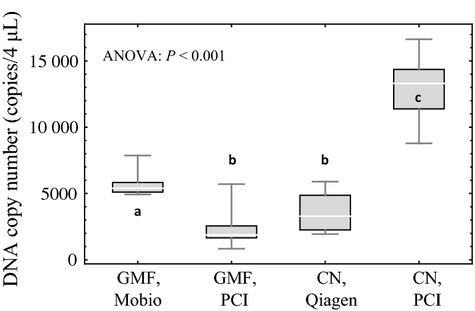
Box and whisker plots for the *PCI-kit comparison experiment*. The top and bottom of the whiskers represent the maximum and minimum values, the top and bottom of the boxes represent the 75% and 25% quartiles, and the lines inside the boxes represent the median values. Significance in pairwise comparisons of treatments is noted by letters a, b and c where different letters represent statistically significant differences between experimental treatments. The four treatments were 1.5-*μ*m glass microfibre filters (GMF) with MoBio extraction, 1.5-*μ*m glass microfibre filters (GMF) with PCI extraction, 0.45-*μ*m cellulose nitrate filters (CN) with Qiagen extraction and 0.45-*μ*m cellulose nitrate filters (CN) with PCI extraction.

All 10 of the samples amplified and were incorporated into the statistical analyses for each of the four experimental treatments (Table1). Tukey's post hoc comparisons of the ANOVA results revealed that the CN filter with PCI extraction yielded significantly more copies of DNA than the other three experimental treatments; the GMF filter with the MoBio extraction yielded significantly more copies of DNA than both the GMF filter with PCI extraction and the CN filter with Qiagen extraction, which were not significantly different from one another (Fig.3).

### DNA extraction experiment

**Fig 4:**
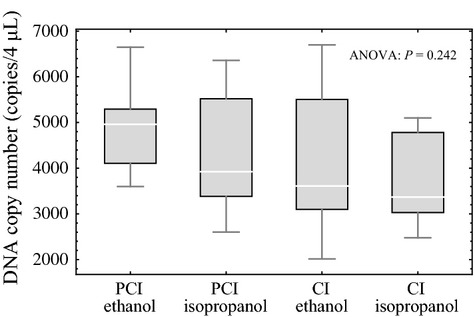
Box and whisker plots for the *DNA extraction experiment*. The top and bottom of the whiskers represent the maximum and minimum values, the top and bottom of the boxes represent the 75% and 25% quartiles, and the lines inside the boxes represent the median values. There was no statistical significance in pairwise comparisons between the four experimental treatments: PCI extraction with ethanol precipitation, PCI extraction with isopropanol precipitation, CI extraction with ethanol precipitation and CI extraction with isopropanol precipitation.

All 10 of the samples amplified and were incorporated into the statistical analyses for each of the four experimental treatments (Table1). Tukey's post hoc comparisons of the ANOVA results revealed no statistically significant differences among the four experimental treatments (Fig.4).

## Discussion

Both preservation buffers, CTAB and Longmire's, successfully preserved filtered eDNA at 20 °C over a 2-week period of time. The DNA copy number increased from the initial time point to the 2-week time point, assuming the results from the fresh treatment indicate the initial copy number in all corresponding experimental treatments. This trend was observed for the filters in the −20 and 45 °C regimes as well. One possible explanation for the observed trend is increased cell lysis efficiency for both buffers with an increase in time. This explanation indicates it is possibly beneficial to leave the filters in the lysis buffer for an extended period of time prior to the DNA extraction. Alternatively, longer incubation times in the 65 °C water bath could significantly increase the yield of extracted DNA as heat facilitates cell lysis and the release of intracellular components, such as DNA (Lu *et al*. 2005). In a comparison between the two buffers, the Longmire's buffer produced a significantly higher DNA copy number than the CTAB buffer for the fresh extractions. In the 45 °C regime, the Longmire's buffer demonstrated a copy number increase from the initial time point to the 1-week time point to the 2-week time point; in comparison, a significant reduction in copy number was observed in the CTAB buffer from the 1-week time point to the 2-week time point, possibly representative of DNA degradation in the elevated storage temperature. These results suggest that while both preservation buffers are adequate for the room temperature preservation of filtered macrobial eDNA, the Longmire's buffer outperformed the CTAB buffer.

The reliance of the PCI extraction approach on chemicals that are harmful to humans is a disadvantage associated with the handling and proper disposal of the phenol and chloroform. When handling these chemicals, certain measures should be considered to reduce the risk of skin contact (i.e. laboratory coat and gloves), eye contact (i.e. safety glasses) or inhalation (i.e. fume hood). It is also recommended to equip laboratories with eyewash stations and safety showers in the event of exposure. In addition to safety measures for handling, the hazardous nature of both chemicals requires them to be disposed of in a manner that conforms to the safety regulations as governed by the organization with which any laboratory is affiliated. One viable alternative for researchers that are interested in using either the CTAB or Longmire's preservation buffers without relying on phenol and chloroform for the DNA extraction is the integration of the room temperature preservation buffer with Qiagen's DNeasy® Blood and Tissue kit (Herath *et al*. 2010; Miller *et al*. 2010).

On the other hand, the PCI approach provides several advantages. First, the PCI extraction protocol potentially yields significantly more copies of targeted eDNA fragments, as demonstrated by the use of 0.45-*μ*m CN filters with both Qiagen's DNeasy® Blood and Tissue kit and the PCI protocol. It is noteworthy that a number of recommended modifications of the DNeasy® protocol exist for increasing the detection probability of targeted species, including the addition of Qiagen's QIAshredder and increasing incubation times (Goldberg *et al*. 2011). These modifications could potentially provide more comparable results to those achieved in the current study with the CTAB preservation buffer and PCI DNA extraction. In the comparison between MoBio's PowerWater® DNA Isolation Kit and the PCI protocol with the 1.5-*μ*m GMF filters, however, the commercial kit yielded significantly more eDNA than the PCI protocol. The MoBio kit, as implemented in the current study, shreds apart the GMF filters through a bead-beating step, exposing the internally captured material (as accomplished by GMF filters through a tortuous path) and possibly increasing lysis efficiency. These results suggest that the addition of a bead-beating step should be considered for the use of the GMF filters with the preservation buffer and PCI extraction.

Second, the PCI approach is substantially cheaper than the commercial kits. Potential per sample costs for eDNA extraction with MoBio's PowerWater® DNA Isolation kit are over $8 (USD), for Qiagen's DNeasy® Blood and Tissue kit are over $2 (USD) and for the PCI extraction protocol are <$0.20 (USD). These values are rough estimates as a number of factors can impact their calculation, but the relative difference in costs between methods is well represented. And finally, the reagents used in the process are well understood, in the public domain, and easily tested and adapted to individual research projects. The lack of statistical significance between the approaches employed in the current study (DNA extraction with and without the use of phenol and DNA precipitation with both ethanol and isopropanol) suggests that researchers have flexibility with the extraction protocols while producing comparable results.

The PCI extraction protocol was successful for all four evaluated filter membrane types: cellulose nitrate (CN), polyethersulfone (PES), polycarbonate track-etch (PCTE) and glass microfibre (GMF). The discrepancy between membrane types in the amount of DNA extracted can, in part, be attributed to differences in pore size. A consistent relationship between pore size and amount of DNA present in the extract has been previously demonstrated (Liang & Keeley 2013), with a measurable decrease in DNA recovery with an increase in pore size (see Fig. S1, Supporting information for independent confirmation of this relationship). The one comparison between membrane types with an equal stated pore size, 0.8 *μ*m CN and 0.8 *μ*m PES, produced statistically comparable copies of targeted eDNA. This relationship differs from previous observations by Liang & Keeley (2013), where mixed cellulose ester filters recovered between 2.6 and 3.9 times more copies of plasmid DNA than PES filters. In addition to potential differences between the type of cellulose filter utilized (mixed cellulose esters comprises both cellulose nitrate and cellulose acetate), the current study may also highlight the complexity of an eDNA sample. Only a singular contributor to eDNA yields (free-floating, extracellular DNA) was evaluated by Liang & Keeley (2013). The increase in relative capture efficiency of the PES filters in the current study may highlight differences between filter types in the potential capture efficiencies of other sources of eDNA, such as those that are intracellular and/or extracellular but bound to other material in the environment (Siuda & Gude 1996; Converse *et al*. 2012; Thomsen *et al*. 2012a), and more closely reflect macrobial eDNA capture potentials in aquatic systems where free-floating DNA is a minority contributor to total eDNA (Turner *et al*. 2014).

The burgeoning field of macrobial eDNA research has already produced noteworthy results in both freshwater and marine environments, including the detection of fish (Thomsen *et al*. 2012a,b; Jerde *et al*. 2013; Kelly *et al*. 2014), amphibians (Dejean *et al*. 2012; Olson *et al*. 2012; Thomsen *et al*. 2012a; Pilliod *et al*. 2013, 2014), reptiles (Piaggio *et al*. 2014), insect larvae and crustaceans (Thomsen *et al*. 2012a; Deiner & Altermatt 2014), mammals (Foote *et al*. 2012; Thomsen *et al*. 2012a) and molluscs (Goldberg *et al*. 2013; Deiner & Altermatt 2014). And as the field continues to grow, the application of next-generation sequencing platforms opens avenues into biodiversity estimates on a large scale (Thomsen *et al*. 2012a) and further integration into the conservation and management of natural resources (Lodge *et al*. 2012). The main goal of this study was to evaluate eDNA preservation and extraction for filtered macrobial environmental DNA, with the potential broad application for studies in a variety of aquatic environments. The Longmire's preservation buffer provides researchers with a room temperature storage buffer that adequately handles elevated temperatures (up to 45 °C in the current study), and the assimilation of the Longmire's preservation buffer into a PCI DNA extraction protocol has the potential to simultaneously reduce per sample costs and increase the recovery of targeted eDNA fragments. The resulting increase in detection probabilities for rare species will benefit future eDNA research for both species-specific assays and large-scale biodiversity estimates.

## Data Accessibility

DNA copy numbers from qPCR plates are available as Data S1 (Supporting information).

## Supporting Information

**Data S1** DNA copy numbers from qPCRs for all four experiments.

**Fig. S1** A total of 40-250 mL water samples were each filtered through single PCTE filters, 10 each for four different pore sizes: 1, 3, 8 and 20 µm.
