# Supplementary material for: The room temperature preservation of filtered environmental DNA samples and assimilation into a phenol–chloroform–isoamyl alcohol DNA extraction
Source: Mol Ecol Resour. 2014 Jun 11;15(1):168–76. doi: 10.1111/1755-0998.12281 (PMC4312482; doi:10.1111/1755-0998.12281)
Supplement: Supplementary file 2 — Fig. S1 A total of 40-250 mL water samples were each filtered through single PCTE filters, 10 each for four different pore sizes: 1, 3, 8 and 20 µm. [file men0015-0168-sd2.docx]

**Supplementary Fig. 1** A total of 40-250mL water samples were each filtered through single PCTE filters, 10 each for four different pore sizes: 1µm, 3µm, 8µm, and 20µm. The box and whisker plots show copy number differences for the targeted bluegill fragments recovered with each filter pore size. The top and bottom of the whiskers represent the maximum and minimum values, the top and bottom of the boxes represent the 75% and 25% quartiles, and the lines inside the boxes represent the median values. Significance in pair-wise comparisons of treatments is noted by letters **a**, **b**, and **c**, where different letters represent statistically significant differences.

**
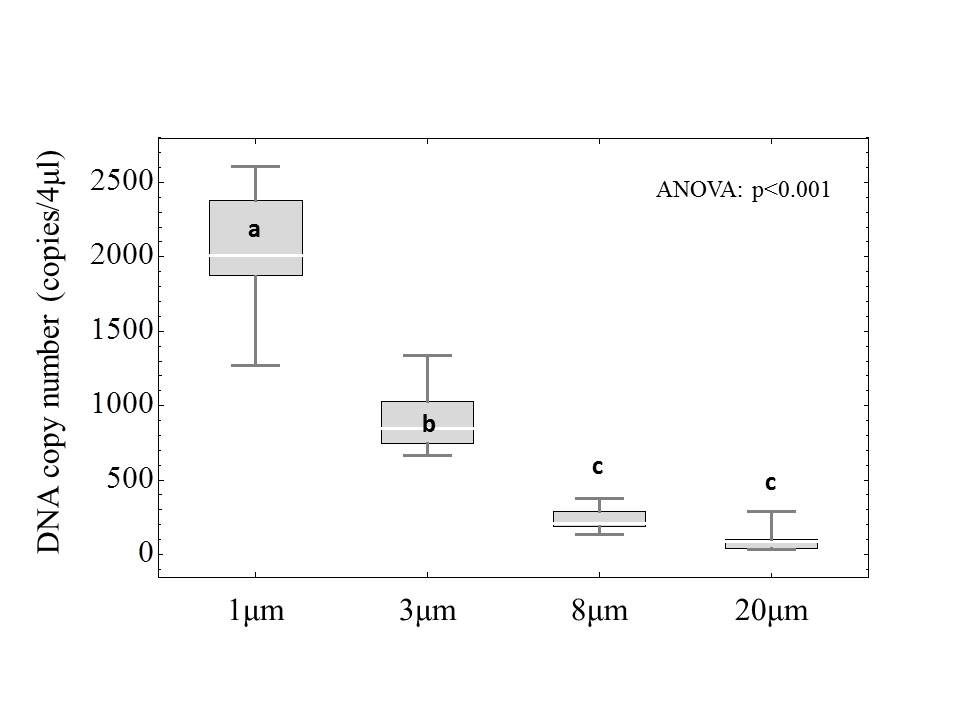
**
